# *In silico *identification of a multi-functional regulatory protein involved in Holliday junction resolution in bacteria

**DOI:** 10.1186/1752-0509-6-S1-S20

**Published:** 2012-07-16

**Authors:** Yan Zhang, Jie Lin, Yang Gao

**Affiliations:** 1 Key Laboratory of Systems Biology, Shanghai Institutes for Biological Sciences, Chinese Academy of Sciences, Shanghai, 200031 China; 2 Computer Network Information Center, Institute of Basic Medical Sciences, Chinese Academy of Medical Sciences and Peking Union Medical College, Beijing, 100005 China

## Abstract

**Background:**

Homologous recombination is a fundamental cellular process that is most widely used by cells to rearrange genes and accurately repair DNA double-strand breaks. It may result in the formation of a critical intermediate named Holliday junction, which is a four-way DNA junction and needs to be resolved to allow chromosome segregation. Different Holliday junction resolution systems and enzymes have been characterized from all three domains of life. In bacteria, the RuvABC complex is the most important resolution system.

**Results:**

In this study, we conducted comparative genomics studies to identify a novel DNA-binding protein, YebC, which may serve as a key transcriptional regulator that mainly regulates the gene expression of RuvABC resolvasome in bacteria. On the other hand, the presence of YebC orthologs in some organisms lacking RuvC implied that it might participate in other biological processes. Further phylogenetic analysis of YebC protein sequences revealed two functionally different subtypes: YebC_I and YebC_II. Distribution of YebC_I is much wider than YebC_II. Only YebC_I proteins may play an important role in regulating RuvABC gene expression in bacteria. Investigation of YebC-like proteins in eukaryotes suggested that they may have originated from YebC_II proteins and evolved a new function as a specific translational activator in mitochondria. Finally, additional phylum-specific genes associated with Holliday junction resolution were predicted.

**Conclusions:**

Overall, our data provide new insights into the basic mechanism of Holliday junction resolution and homologous recombination in bacteria.

## Background

Homologous recombination is a fundamental mechanism in biology that rearranges genes within and between chromosomes, promotes DNA repair, and guides segregation of chromosomes at division. This process is common to all forms of life and involves the exchange (i.e., breakage and reunion) of DNA sequences between two chromosomes or DNA molecules [1-4]. Such exchange provides a valid evolutionary force that contributes to promote genetic diversity and to conserve genetic identity. In addition, homologous recombination is also used in horizontal gene transfer to exchange genetic material between different strains and species of bacteria and viruses [5].

Although homologous recombination varies widely among different organisms and cell types, most forms of it involve the same basic steps: (i) after a DNA break occurs, sections of DNA around the break on the 5' end of the damaged chromosome are removed in a process called resection; (ii) in the strand invasion step that follows, an overhanging 3' end of the damaged chromosome then "invades" an undamaged homologous chromosome; (iii) after strand invasion, one or two cross-shaped structures (called Holliday junctions) are formed to connect the two chromosomes. Holliday junction (or four-way junction) has been generally assumed as a key intermediate in genetic recombination and DNA repair since its discovery in 1964 [6]. They are highly conserved structures from prokaryotes to mammals, which adjoin two DNA duplexes, forming a branch point where four helices are interconnected by strand exchange [7,8].

Because Holliday junctions provide a covalent linkage between chromosomes, their efficient resolution is essential for proper chromosome segregation. Enzymes that resolve Holliday junctions by endonucleolytic cleavage have been isolated from bacteriophages, bacteria, archaea and certain eukaryotes [9-12]. In *Escherichia coli*, the enzymes that are involved in resolution of Holliday junction include RuvABC, RecU, RecG, and RusA [13-15]. The RuvABC proteins (or RuvABC resolvasome) constitute a simple and the most widely used system for the processing of Holliday junctions. RuvAB proteins catalyze the branch migration whereas RuvC endonuclease resolves the Holliday junction into duplex products [15,16]. RecU, a RuvC functional analog, was found to serve as a Holliday junction resolvase in some firmicutes and mollicutes that lack RuvC [17,18]. The RecG protein is a DNA helicase and may promote branch migration of a variety of branched DNAs including Holliday junctions [19,20]. The RusA protein is a homodimeric Holliday junction-specific endonuclease and can bind a variety of branched DNA structures [21,22]. RecG may be required by RusA to branch migrate Holliday junctions to cleavable sequences [9].

Homologs of RuvABC, RecU, RecG, and RusA are absent from almost all sequenced archaea and eukaryotes. In archaea, the Hjc protein, a distantly related member of the type II restriction endonuclease family, has been characterized to serve as a Holliday junction resolving enzyme [23,24]. Little is known about the mechanism of eukaryotic Holliday junction resolution and the enzymes involved. It was reported that *Saccharomyces cerevisiae *contains a Holliday junction resolvase Cce1 [25,26], an equivalent enzyme from *Schizosaccharomyces pombe *(named Ydc2) has also been found [27]. These enzymes are targeted to the mitochondria, suggesting that they can only cleave junctions formed during recombination of mitochondrial DNA. Very recently, a nuclear Holliday junction resolvase was first identified from both humans and yeast [28]. These resolvases (GEN1 in human and its yeast ortholog Yen1) represent a new subclass of the the Rad2/XPG family of nucleases, and promote Holliday junction resolution in a manner similar to that shown by the *E. coli *RuvC [29,30]. However, the precise mechanism regulating the activities of these enzymes is unknown and the factors involved remain unidentified.

In this study, we carried out comparative genomics approaches to investigate the mechanisms of Holliday junction resolution in prokaryotes. Occurrence of known components of Holliday junction resolution (e.g., RuvABC and RecU) could be easily identified by comparative genomics. Our analysis also generated evidence for a novel DNA-binding regulatory protein family involved in Holliday junction resolution in bacteria. Homologs of this family were detected in a variety of eukaryotes and are predicted to be localized in mitochondria. Overall, these data provide new insight for better understanding the basic mechanism of homologous recombination in nature.

## Results and discussion

### Distribution of the RuvABC/RecU Holliday junction resolution system in prokaryotes

Except a very small number of organisms (less than 2%) with small and condensed genomes (mostly parasites), all sequenced bacteria contain RuvA and RuvB genes. As RuvAB complex may catalyze both Holliday junction branch migration and replication fork reversal [31,32], the occurrence of their genes may not precisely reflect the Holliday junction resolution trait. Thus, we used the co-occurrence of RuvABC or RuvAB/RecU as a signature for the presence of RuvABC/RecU-dependent Holliday junction resolution trait.

**Figure 1:**
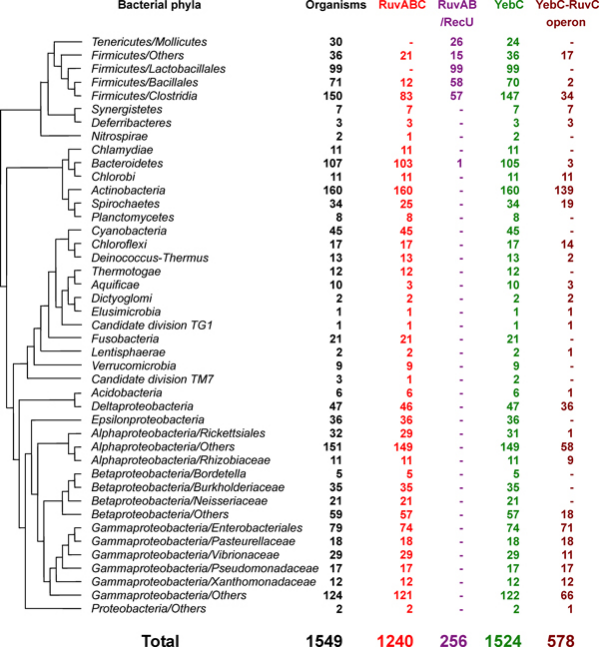
**Occurrence of RuvABC system and YebC family in different bacterial taxa**. The tree is based on a highly resolved phylogenetic tree of life (see Methods). "YebC-RuvC operon" represents organisms in which YebC and RuvC genes are located within the same operon.

Sequence analysis of bacterial genomes revealed a wide distribution of RuvABC resolvasome. We identified 1240 organisms (80% of all sequenced bacteria) that contain this system, which is consistent with previous observations that RuvABC complex is the most important Holliday junction resolution system in bacteria [9,33]. Details are shown in Table S1 [see additional file 1]. All RuvC-containing organisms have RuvA and RuvB, most of which have RuvABC genes within the same operon. The RuvAB/RecU system was detected in 256 organisms (255 in *Firmicutes *and *Mollicutes*), almost all of which lack RuvC genes. All detected RecU genes are distant from RuvAB genes based on genomic context analysis. Among all examined genomes, only four organisms belonging to *Firmicutes/Clostridia *were found to have both RuvC and RecU genes. Figure 1 shows the distribution of RuvABC and RuvAB/RecU systems in different bacterial taxa based on a highly resolved phylogenetic tree of life developed by Ciccarelli and coworkers [34]. Our data are consistent with previous studies that RuvC was replaced by its functional analog RecU in firmicutes and mollicutes [17,18]. On the other hand, the absence of both resolvase genes in 27 organisms that have RuvAB complex might suggest the presence of unknown resolvase or alternative resolution system (such as RecG-RusA) in these organisms.

In contrast to bacteria, only two closely related archaea in *Methanomicrobiales *(*Methanoregula boonei *and *Methanospirillum hungatei*) were found to have RuvABC system, suggesting that they recently acquired this system from bacteria by horizontal gene transfer. No RecU homolog could be detected in archaea.

### Identification of a new family involved in RuvABC-dependent Holliday junction resolution in bacteria

Since the RuvABC complex has been shown to be the most widely used system for the resolution of Holliday junctions in bacteria, identification of functional linkages involving RuvABC (especially RuvC which is specific for Holliday junction resolution trait) may help understand the details of this important process. First, we used STRING web server [35] to examine possible functional linkages based on neighborhood, gene fusion and co-occurrence analyses. The top candidates for RuvA, RuvB or RuvC are shown in Table 1. Except for known components of RuvABC, the protein hit with the best score was YebC, a putative cytoplasmic protein with unassigned function (COG0217, uncharacterized conserved protein; pfam01709, domain of unknown function DUF28). This gene was located very close to or even within the same operon with RuvC in many bacteria. In addition, YebC and RuvABC showed similar patterns of occurrence in most bacterial phyla based on the STRING output. The next predicted RuvABC link was YbgC, a bacterial 4-hydroxybenzoyl-CoA thioesterase involved in phospholipid metabolism and is also associated with the Tol-Pal system [36]. Most of other candidates predicted by STRING are also involved in Tol-Pal system. It has been known that this system is important for cell envelope integrity and is part of the cell division machinery. In *E. coli*, the Tol-Pal system is composed of the YbgC, TolQ, TolA, TolR, TolB, Pal and YbgF proteins [36,37]. So far it is unclear whether some of these proteins are involved in DNA repair and recombination. Similar analysis was also done for RecU and no strong functional partners could be assigned (data not shown). In this study, we only focus on YebC proteins.

**Table 1:** STRING analysis of genes functionally associated with RuvABC resolvasome.

Rank	RuvA	RuvB	RuvC
1	RuvB	RuvA	RuvB
2	RuvC	RuvC	RuvA
3	YebC	YebC	YebC
4	YbgC	QueA	YbgC
5	TolB	YbgC	YeeN
6	QueA	TolB	TolB
7	FolC	PanB	CysS
8	MaeB	TolQ	TolQ
9	YeeN	TolR	QueA
10	TolQ	YjeS	PurH

Considering that YebC might be functionally associated with RuvABC resolvasome, we further analyzed the distribution of this protein family in all sequenced prokaryotes. Homologs of YebC were not detected in archaea, implying that YebC may either have evolved in bacteria or lost in the ancestors of archaea. In bacteria, the distribution of YebC appeared to be wider than RuvABC system (Figure 1). Almost all sequenced organisms (98%) possess YebC genes, suggesting that YebC may be also involved in other processes independent of RuvABC system. However, the facts that all RuvC-containing organisms have YebC, and that YebC and RuvC genes are located in the same operon in approximately half of the RuvC-containing organisms (Figure 1), indicate a strong relationship between them. These results were consistent with a previous study of some "hypothetical" genes expressed in *Haemophilus influenzae*, which also suggested a potential association of YebC with RuvABC in this organism [38].

### Phylogeny and functional classification of YebC family

**Figure 2:**
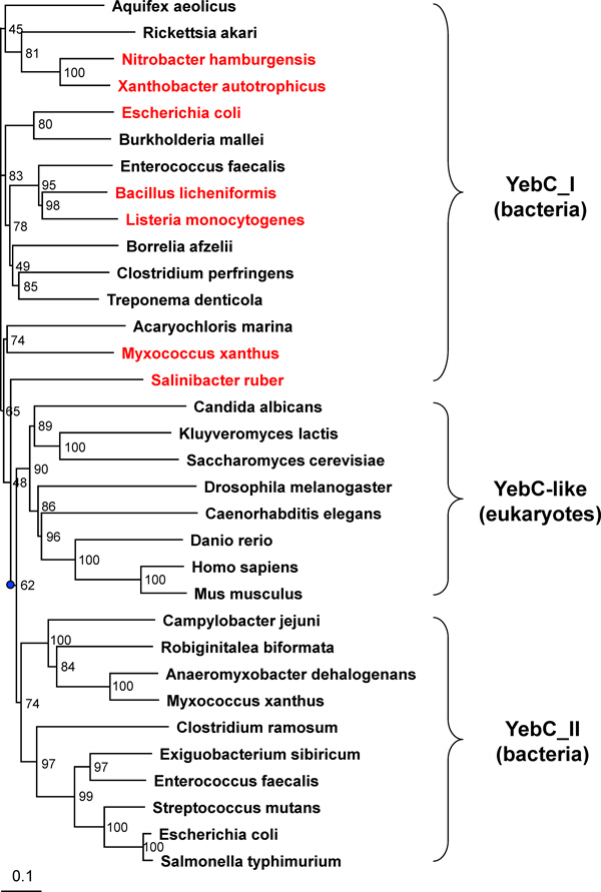
**Phylogenetic analysis of YebC family**. Organisms where YebC genes are located very close to RuvABC genes are shown in red. The root node of the tree is shown as a blue dot. Separate branches for the two subtypes of YebC in bacteria and the eukaryotic YebC-like proteins are also shown. Both bootstrap support (the number of times each branch was supported in bootstrap replication) and the measurement of distance for the branch lengths (shown by a bar) are indicated.

The majority of YebC-containing bacteria (92%) have single copy of this gene. Intriguingly, in the organisms that have more than one YebC homologs, there is always one protein whose gene is located very close to either RuvC or RuvAB (when RuvC is absent) genes, implying that YebC proteins could be divided into different subgroups. Phylogenetic analysis of YebC proteins from sequenced bacteria revealed that YebC family may contain two subtypes: YebC_I and YebC_II (Figure 2).

**Figure 3:**
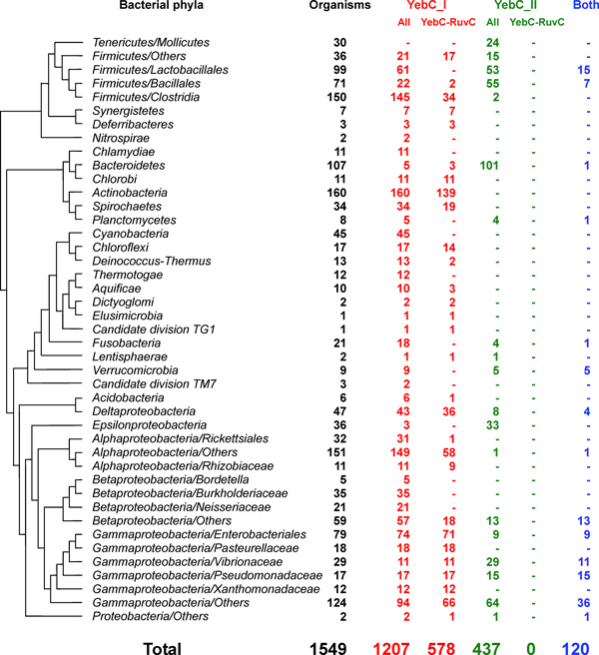
**Occurrence of two YebC subfamilies in bacteria**. YebC_I, organisms that contain members of YebC_I subfamily; YebC_II, organisms that contain members of YebC_II subfamily; Both, organisms that contain members of both YebC_I and YebC_II subfamilies; YebC-RuvC, organisms in which YebC_I/YebC_II and RuvC genes are located within the same operon.

Further analysis of bacterial genomes revealed wide but unbalanced distribution of different YebC subfamilies (Figure 3). YebC_I was present in nearly all bacterial phyla with the exception of *Mollicutes*, whereas YebC_II was detected in approximately half of the examined phyla (mostly in *Bacteroidetes*, *Firmicutes *and *Proteobacteria*). This observation suggests that YebC_I proteins may be used by most bacterial lineages and should be involved in an ancient trait that was common to all or almost all species in this domain of life. Interestingly, only members of YebC_I subgroup were found to be located very close to the genes encoding RuvC (Figure 3), implying a strong association between YebC_I and RuvC. In some of the organisms that lack RuvC (no matter whether they have RecU genes or not), YebC_I was located next to RuvAB genes, suggesting a potential link between YebC_I and RuvAB complex in the absence of RuvC in these organisms. However, it is unclear if YebC_I is functionally related to RecU. In contrast, there is no evidence that YebC_II subgroup might be involved in RuvABC-dependent Holliday junction resolution, even though only YebC_II members were observed in most *Bacteroidetes*, *Epsilonproteobacteria *and *Gammaproteobacteria/Vibrionaceae *that have RuvABC resolvasome. Thus, it appeared that YebC_I proteins are functionally associated with RuvABC resolution system. YebC_II might have evolved from YebC_I proteins with novel function. It should be noted that several YebC_I proteins were also found in a small number of organisms that lacked the complete RuvABC or RuvAB/RecU system, implying that YebC_I might have additional function in these organisms.

**Figure 4:**
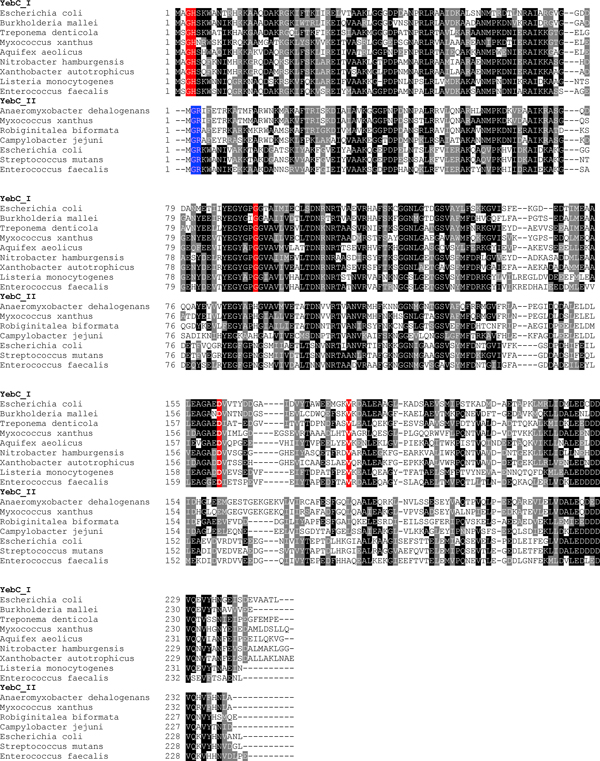
**Multiple sequence alignment of YebC proteins in bacteria**. Representative sequences were divided into YebC_I and YebC_II subgroups. Residues which are strictly conserved in the YebC_I subgroup are shown in red background. Residues which are strictly conserved in the YebC_II subgroup are shown in blue. Other residues shown in white on black or grey are conserved in homologs.

Multiple alignment of YebC_I and YebC_II sequences suggested several specific residues which are only present in each subfamily (Figure 4). An attractive hypothesis is that YebC_I is functionally different from YebC_II, perhaps distinguished by some of these conserved residues.

### Prediction of the function of YebC proteins

Although YebC is a large family of widespread conserved proteins whose function is unknown, this group of proteins has been extensively characterized from the structural perspective. To date, the crystal structures of YebC proteins from *Aquifex aeolicus *(YebC_I), *E. coli *(YebC_I), and *Helicobacter pylori *(YebC_II) have been solved (PDB ID codes 1LFP, 1KON, and 1MW7, respectively). A previous structural analysis of *A. aeolicus *YebC revealed a large cavity with a predominance of negatively charged residues on the surface of this protein [39]. Interestingly, all three structure-solved proteins have a putative DNA binding function, suggesting that YebC proteins may serve as a potential transcription factor. A recent study reported that the YebC protein in *Pseudomonas aeruginosa *(PA0964, YebC_I) may be involved in negatively regulating the quorum-sensing response regulator pqsR of the PQS system by binding at its promoter region [40]. This result implied the complexity of the function of YebC in nature.

Although the function of YebC proteins and the biological pathways they are involved in are unclear, our current studies provide some useful information for this widely used protein family: (i) both YebC_I and YebC_II subgroups may bind DNA; (ii) YebC_I proteins may serve as a multi-functional transcription regulator mainly involved in regulating the expression of RuvABC genes as well as other genes such as pqsR; (iii) YebC_II might have evolved from YebC_I by gene duplication and have novel function independent of Holliday junction resolution or even DNA recombination. A future challenge would be to understand the DNA binding patterns of YebC_I and YebC_II proteins as well as additional processes they may regulate.

### Investigation of YebC-like proteins in eukaryotes

Significant YebC homologs were also detected in a variety of eukaryotes, including fungi, plants and animals (Table S2 [see additional file 2]). Very recently, it was reported that a mutation in the human gene encoding a YebC homolog (named CCDC44, localized to the mitochondria) led to a specific defect in the synthesis of the mitochondrial DNA-encoded cytochrome c oxidase subunit I (COX I) [41]. Thus, the human CCDC44 protein was renamed as TACO1, which may serve as a mammalian mitochondrial translational activator of COX I. Possible mechanisms of TACO1 action to ensure translation of COX 1 were also considered: (i) securing an accurate start of translation; (ii) stabilizing the elongating polypeptide; and (iii) interacting with the peptide release factor [41,42].

We analyzed the sequences of all eukaryotic YebC-like proteins and the evolutionary relationship with their bacterial counterparts. All detected YebC-like proteins in eukaryotes have mitochondrial signal sequences, suggesting that they are mitochondria-targeted proteins. Phylogenetic analysis of bacterial YebC and eukaryotic YebC-like proteins showed that the eukaryotic YebC-like proteins were clustered with YebC_II subfamilies (Figure 2), implying that these YebC-like proteins (including human TACO1) might have evolved from ancient YebC_II proteins. The mitochondrial signal sequences were then added to target them into the mitochondria as a specific translational activator, at least in metazoan mitochondrial genome. As eukaryotes lack the RuvABC resolvasome, it is unclear whether these YebC-like proteins are involved in homologous recombination in mitochondria, or whether they still have the capacity to bind mitochondrial DNA. Further studies are required to determine the substrates and function of YebC-like proteins in other organisms as well as their relationship with DNA repair and recombination in mitochondria.

### Prediction of additional phylum-specific genes associated with RuvABC resolvasome

Comparative genomics studies also suggested additional candidate genes involved in RuvABC-dependent Holliday junction resolution in certain bacterial phyla. In *Firmicutes/Clostridia*, most organisms possess a conserved hypothetical protein (CTC02214 in *Clostridium tetani*, a distant homolog of pfam08955, BofC C-terminal domain) whose gene is always located next to either YebC or RuvC gene, implying a potential functional link with them. However, orthologs of this protein family were exclusively detected in *Clostridia*, suggesting that this protein might be newly evolved in this phylum. Similarly, another conserved hypothetical protein (DUF208 super family; COG1636, uncharacterized protein conserved in bacteria) was also identified in a variety of distantly related organisms where its gene is often located close to either YebC or RuvABC genes (data not shown). Further studies, however, are needed to verify their function and the relationship between these genes and genetic recombination in bacteria.

## Conclusions

In this study, we carried out comparative genomics to identify a novel DNA-binding regulatory protein family, YebC, which was strongly linked to Holliday junction resolution in bacteria. Phylogenetic analysis revealed that YebC might be divided into two functionally different subgroups: YebC_I and YebC_II. YebC_I may serve as a multi-functional transcriptional regulator that mainly regulates the gene expression of RuvABC resolvasome in bacteria. It could not be excluded that YebC_II is involved in homologous recombination, but current evidence does not provide strong support for this possibility. Further studies on eukaryotic YebC-like proteins suggested that they may have evolved from YebC_II subgroup and have different function to serve as a specific translational activator in mitochondria.

## Methods

### Genomes, sequences and resources

Fully sequenced genomes from the Entrez Genome Database at NCBI were used in this study [43]. Because of the large number of strains for some bacterial species, only one strain was selected for each species. A total of 1549 bacteria, 97 archaea and 330 eukaryotes were analyzed (as of October 2011).

We used *E. coli *RuvA (COG0632, Holliday junction resolvasome DNA-binding subunit), RuvB (COG2255, Holliday junction resolvasome helicase subunit), RuvC (COG0817, Holliday junction resolvasome endonuclease subunit) and *Bacillus subtilis *RecU (pfam03838, recombination protein U) sequences as queries to search for RuvABC or RuvAB/RecU-dependent Holliday junction resolution trait. For each of these proteins, TBLASTN [44] was initially used to identify genes coding for homologs with a cutoff of E-value ≤ 0.1. Orthologous proteins were then defined using the conserved domain (COG/Pfam) database and bidirectional best hits [45].

The STRING (Search Tool for the Retrieval of Interacting Genes/Proteins) database and programs [35] were used to identify gene candidates that may be functionally related to RuvABC resolvasome. Different parameters were used for better performance.

### Multiple sequence alignment and phylogenetic analysis

Sequence alignments were performed with CLUSTALW [46] using default parameters. Ambiguous alignments in highly variable (gap-rich) regions were excluded. The resulting multiple alignments were then checked for conservation of residues and manually edited. Phylogenetic analyses were performed using PHYLIP programs [47]. Pairwise distance matrices were calculated by PROTDIST to estimate the expected amino acid replacements per position. Neighbor-joining trees were obtained with NEIGHBOR and the most parsimonious trees were determined with PROTPARS.

## List of abbreviations

COX I: cytochrome c oxidase subunit I; STRING: Search Tool for the Retrieval of Interacting Genes/Proteins.

## Competing interests

The authors declare that they have no competing interests.

## Authors' contributions

YZ designed the study. YZ carried out computational studies, including comparative genomics, sequence alignment, phylogenetic analysis and wrote the manuscript. JL and YG analyzed the data and edited the manuscript. All authors read and approved the final manuscript.

## Supplementary Material

Additional file 1 **This file contains the distribution of RuvA, RuvB, RuvC, RecU and YebC genes in sequenced bacteria**. Click here for file

Additional file 2 **This file contains the distribution of YebC-like genes in sequenced eukaryotes**. Click here for file
